# Supplementary material for: End Sequence Analysis Toolkit (ESAT) expands the extractable information from single-cell RNA-seq data
Source: Genome Res. 2016 Oct;26(10):1397–410. doi: 10.1101/gr.207902.116 (PMC5052061; doi:10.1101/gr.207902.116)
Supplement: Supplemental Material [file supp_gr.207902.116_Supp_Materials.tar.gz › ESAT-esat_v0.1/doc/html/functions_func_u.html]

ESAT: Class Members - Functions


ESAT


- Main Page
- Related Pages
- Packages
- Classes
- Files

- Class List
- Class Index
- Class Hierarchy
- Class Members

- All
- Functions
- Variables
- Enumerator

- a
- b
- c
- d
- e
- f
- g
- h
- i
- j
- k
- l
- m
- n
- o
- p
- q
- r
- s
- t
- u
- v
- w
- z


All Classes Namespaces Files Functions Variables Enumerator Pages

### - u -

- uminus()
  : Jama.Matrix
- UnableToFitException()
  : broad.core.siphy.UnableToFitException
- unchunk()
  : broad.core.sequence.FastaSequenceIO
- UnfairDie()
  : broad.core.math.UnfairDie
- ungappedMatchStartOnFirstSequence()
  : umms.core.alignment.SmithWatermanAlignment
- union()
  : broad.core.sequence.SequenceRegion
  , umms.core.annotation.Annotation
  , umms.core.annotation.BasicAnnotation
  , umms.core.annotation.CompoundInterval
  , umms.core.annotation.SingleInterval
- unloadAllSequences()
  : broad.core.sequence.Sequence
- unloadEncodedSequence()
  : broad.core.sequence.Sequence
- unloadSequence()
  : broad.core.sequence.Sequence
  , broad.pda.feature.genome.Chromosome
- UnorderedGenePair()
  : umms.core.alignment.FeatureSequenceAlignment.UnorderedGenePair
- unsetStranded()
  : broad.pda.seq.segmentation.AlignmentDataModel
  , broad.pda.seq.segmentation.GenericAlignmentDataModel
- updateBoundariesWithoutCrossingIntrons()
  : broad.core.util.CollapseByIntersection
- updateDistanceToNeighbors()
  : broad.pda.gene.NeighborAnalysis
- updateGeneByFirstCounts()
  : broad.pda.seq.segmentation.AlignmentDataModel
  , broad.pda.seq.segmentation.GenericAlignmentDataModel
- updateIsoformsNameWithLocusName()
  : broad.pda.annotation.Locus
- updateReadInfoWithBarcode()
  : broad.pda.seq.fastq.FastqSplitterByBarcode
- updateScores()
  : umms.core.model.score.MultiScore
- updateScrToBedScore()
  : broad.pda.annotation.BEDFileParser
  , broad.pda.gene.GeneWithIsoforms
- uploadSetOfFiles()
  : broad.pda.annotation.BEDFileParser
- uppercase()
  : broad.core.sequence.Sequence
- useMergedUn()
  : broad.pda.feature.genome.DirectoryInstalledGenomeAssembly


---

Generated on Fri Aug 15 2014 15:11:17 for ESAT by  

 1.8.7
